# Fabrication of a Gelatin-Based Microdevice for Vascular Cell Culture

**DOI:** 10.3390/mi14010107

**Published:** 2022-12-30

**Authors:** Satoko Sasaki, Tomoko Suzuki, Kyojiro Morikawa, Michiya Matsusaki, Kae Sato

**Affiliations:** 1 Department of Chemical and Biological Sciences, Faculty of Science, Japan Women’s University, 2-8-1 Mejirodai, Bunkyo, Tokyo 112-8681, Japan; 2 Institute of Nanoengineering and Microsystems, Department of Power Mechanical Engineering, National Tsing Hua University, No. 101, Section 2, Kuang-Fu Road, Hsinchu 300044, Taiwan; 3 Collaborative Research Organization for Micro and Nano Multifunctional Devices, The University of Tokyo, 7-3-1 Hongo, Bunkyo, Tokyo 113-8656, Japan; 4 Division of Applied Chemistry, Graduate School of Engineering, Osaka University, 1-1 Yamadaoka, Suita, Osaka 565-0871, Japan

**Keywords:** gelatin, microbial transglutaminase, microfluidics, vascular, cell culture

## Abstract

This study presents a novel technique for fabricating microfluidic devices with microbial transglutaminase-gelatin gels instead of polydimethylsiloxane (PDMS), in which flow culture simulates blood flow and a capillary network is incorporated for assays of vascular permeability or angiogenesis. We developed a gelatin-based device with a coverslip as the bottom, which allows the use of high-magnification lenses with short working distances, and we observed the differences in cell dynamics on gelatin, glass, and PDMS surfaces. The tubes of the gelatin microfluidic channel are designed to be difficult to pull out of the inlet hole, making sample introduction easy, and the gelatin channel can be manipulated from the cell introduction to the flow culture steps in a manner comparable to that of a typical PDMS channel. Human umbilical vein endothelial cells (HUVECs) and normal human dermal fibroblasts (NHDFs) were successfully co-cultured, resulting in structures that mimicked blood vessels with inner diameters ranging from 10 µm to 500 µm. Immunostaining and scanning electron microscopy results showed that the affinity of fibronectin for gelatin was stronger than that for glass or PDMS, making gelatin a suitable substrate for cell adhesion. The ability for microscopic observation at high magnification and the ease of sample introduction make this device easier to use than conventional gelatin microfluidics, and the above-mentioned small modifications in the device structure are important points that improve its convenience as a cell assay device.

## 1. Introduction

Blood vessels are altered by various diseases, such as cancer and hypertension, and assays of angiogenesis and vascular permeability are essential for the evaluation of drug efficacy for the treatment of these diseases. Recently, research on organ-on-a-chip has gained substantial attention owing to their wide range of applications [1,2,3,4,5,6,7,8,9]. “Organ-on-a-chip” or “MPS (Micro physiological systems)” is a system comprising engineered or natural miniature tissues grown inside microfluidic chips. To better mimic the characteristics and functions of specific human organs in vitro, the integration of a perfusable and functional three-dimensional (3D) microvasculature into organ-on-a-chip systems is crucial. Polydimethylsiloxane (PDMS) is the most commonly used material in organ-on-a-chip device fabrication. However, it has poor permeability [10] and is stiffer than biological tissue, rendering the cell culture environment in a PDMS device different from that in an in vivo environment. Moreover, in PDMS devices with narrow channels, the microfluidic culture medium provided to the cells is less than that provided in a culture dish; therefore, they require frequent medium exchange, making long-term culture challenging [7].

In contrast, gelatin, a degradation product of collagen, a type of extracellular matrix (ECM), has been utilized in the field of tissue engineering and as a scaffold for cell sheets for transplantation [11]. Gelatin is an excellent material because it is inexpensive and readily available. Reports have shown three-dimensional muscle-like tissues or cardiac-like tissues with specific layer orientations and interlayer angles created via stripe structures on coverslips with cross-linked gelatin [12,13,14,15,16,17,18,19,20]. Three-dimensional vascularized tissues have also been fabricated via three-dimensional bioprinting of gelatin-based ink [21,22,23]. In this study, we propose microdevices that more closely resemble the biological environment by incorporating tissue engineering techniques using gelatin.

In the last decade, ECM hydrogels have been integrated into PDMS microchannels to develop organ-on-a-chip vascular and lymphatic models [24,25,26,27]. Gong et al. have reported that lymphatic endothelial cells with a diameter of 250 µm were constructed inside collagen gels in a PDMS channel [27]. Microdevices constructed using hydrogels without PDMS have also been reported. An example of hydrogel-based microfluidics is an alginate gel-based device with a channel that is 100 µm deep and 200 µm wide [28]. Moreover, devices containing microchannels constructed with gelatin have been reported [28,29,30,31,32,33,34,35,36,37]; however, microfluidic devices fabricated with gelatin gels are not yet commonly used because they are more difficult to process than PDMS. Nonetheless, in gelatin-based microdevices, immersion of the device in a culture medium allows for the removal of waste products from the cells and the replenishment of nutrients from the culture medium. This is an advantage over PDMS devices with embedded gelatin microchannels. Therefore, microfluidic devices made entirely with gelatin may be in high demand.

To use gelatin for cell culture, crosslinking is necessary to prevent gelatin dissolution at 37 °C because gelatin gels cannot maintain their structure above 30 °C. In cell culture devices with microstructures, gels with many crosslinking points are necessary to maintain the structure. Three types of substances have been used as crosslinkers for gelatin gels, including microbial transglutaminase (mTG) [19], which is an enzyme that links glutamine and lysine residues; genipin [38], which is a plant-derived aldehyde compound, and methacrylic acid [39], which is bound by radical reactions. A comparison between these three substances is shown in Table 1. Makita et al. have fabricated grooves, holes, and pillars with widths or diameters of 2 µm, 1 µm, or 500 nm with crosslinked gelatin with genipin [38]. Moreover, gelatin methacrylate (GelMA) has been widely used in three-dimensional bioprinting as a bio-ink to support cells [39]. Hasan et al. have fabricated tri-layer biomimetic blood vessel-like structures with fibroblasts, smooth muscle cells, and vascular endothelial cells on a microfluidic platform using GelMA-hydrogel [40]. However, GelMA is expensive, whereas genipin is superior in terms of immediate fabrication and cost-effectiveness. Although it is a crosslinking agent with low cytotoxicity, genipin is an active extract of traditional Chinese medicine that has several bioactivities [41]. In addition, during the gelling process, genipin crosslinks amino groups, causing blue pigment formation [38,42], which interferes with fluorescence observation. Therefore, it is not advisable to use genipin as a substrate in cell culture. Paguirigan et al. [29] have successfully fabricated a gelatin cell culture channel using a mixture of mTG and gelatin by pouring the mixture into a mold. The mTG crosslinks with gelatin to form a cytocompatible hydrogel [20,43] within a reasonable time frame (from 5–20 min for gel formation) [44] without the toxicity of any chemical crosslinker, and the reaction occurs in a more physiological-like environment [45]. Overall, mTG is the most suitable crosslinker for the fabrication of gelatin microchannels.

This study aimed to develop a technique for fabricating microfluidic devices with mTG-gelatin gels instead of PDMS, in which flow culture simulates blood flow and a capillary network is incorporated for assays of vascular permeability or angiogenesis. We developed a gelatin-based device using a coverslip as the bottom to allow the use of high-magnification lenses with short working distances and using tubes designed to be difficult to pull out of the inlet hole of the gelatin channel. We observed differences in cell dynamics on gelatin, glass, and PDMS surfaces with this device. The device enabled microscopic observation at high magnification, and the difficulty of pulling the tubes out of the inlet hole of the gelatin channel during pipetting facilitated culture and observation of vascular endothelial cells more easily than conventional gelatin microfluidic channels. These small modifications in the device structure are important points that improve its convenience as a cell assay device.

## 2. Materials and Methods

### 2.1. Modification of Coverslips with APTES-Glutaraldehyde (GA)

Glass coverslips were cleaned using 0.1 M NaOH for 5 min. The cleaned coverslips were modified using 0.5% 3-aminopropyltriethoxysilane (APTES; Tokyo Kasei Kogyo, Tokyo, Japan) in ethanol for 5 min, followed by modification with 0.5% GA (1st grade; FUJIFILM Wako Pure Chemical, Osaka, Japan) in deionized water for 30 min. After modification, the coverslips were washed with deionized water and then dried at 100 °C for 5 min.

### 2.2. Fabrication of PDMS Molds

The SU-8 master molds were fabricated using standard microfabrication techniques [3] with minor modifications. A silicon wafer (Mitsubishi Materials Trading, Tokyo, Japan) was cut into 24 mm × 24 mm squares using a diamond cutter. The substrates were ultrasonically cleaned in 2-propanol for 2 min, followed by 2 min in deionized water, and dried at 100 °C for 10 min. Further organic residues were oxidized with oxygen plasma at 100 W for 2 min using an air-plasma generator (CUTE FC-10029; Femto Science, *Hwaseong-si,* Gyeonggi-Do, Republic of Korea).

The photoresist SU-8 3050 (0.15 g for 100 µm deep channel or 0.20 g for 200 µm deep channel; Nippon Kayaku, Tokyo, Japan) was poured and uniformly distributed on the silicon wafer. The coated photoresist was prebaked at 95 °C for 30 min (100 µm deep channel) or 45 min (200 µm deep channel), exposed to UV irradiation for 30 s (MJB3 Mask aligner; SUSS MicroTec, Garching bei München, Germany) through a photomask film, post-baked at 65 °C for 1 min, followed by 10 min at 95 °C, developed with ethyl lactate (1st Grade, FUJIFILM Wako Pure Chemical, Osaka, Japan) for 1 min, and rinsed with 2-propanol. The microchannel pattern was concave in the SU-8 mold. The depth and width of the microchannel pattern in the mold were measured using a microscope (STM6; OLYMPUS, Tokyo, Japan). The SU-8 mold was subsequently used to fabricate the PDMS mold.

A prepolymer of PDMS (SILPOT 184; Dow Corning, Midland, MI, USA) and a curing agent were mixed in a 10:1 weight ratio and placed onto the SU-8 master in a plastic dish to hold the prepolymer. The PDMS mixture was cured in an oven at 65 °C for 60 min, and the cured PDMS was peeled off from the master, placed on a glass slide, and cured at 100 °C for 60 min. The microchannel pattern was convex in the PDMS mold.

### 2.3. Fabrication of Crosslinked Gelatin Microfluidic Devices

The crosslinked gelatin devices were fabricated based on a report by Paguirigan et al. [29]. The gelatin/mTG prepolymer mixture was prepared using 11 wt% gelatin (type A from porcine skin, 500G; Nitta Gelatin, Osaka, Japan) solution in phosphate-buffered saline (PBS) (−). The gelatin solution was heated at 37 °C for 30 min to solubilize the gelatin, and 0.02% chloroform was added to sterilize the solution. mTG (1000 U/g), kindly provided by Ajinomoto (Tokyo, Japan), was dissolved in PBS (−) to obtain a 10 U/mL solution. The final concentrations in the gelatin mixture were 10 wt% gelatin and 1 U/mL mTG.

**Figure 1:**
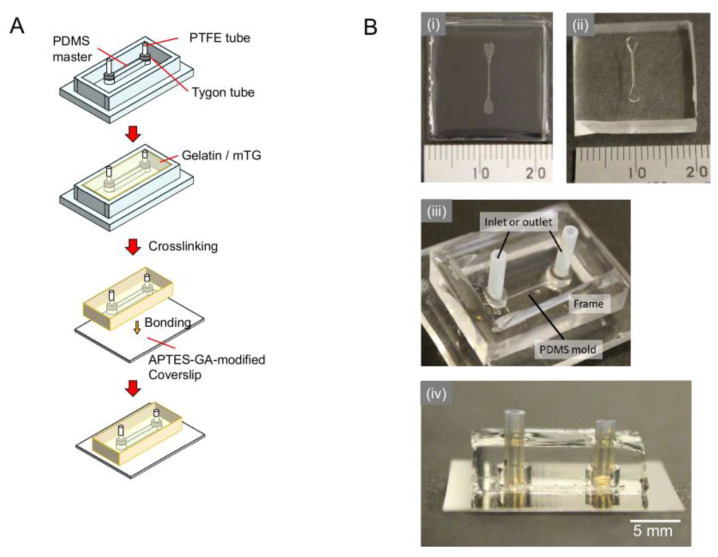
The fabrication process of the gelatin microfluidic device: (**A**) schematic illustration of the fabrication process; (**B**) photograph of (**i**) SU-8 mold, 200 μm width × 10 mm length × 300 μm height; (**ii**) PDMS mold; (**iii**) inlet tubes placed on PDMS mold with PDMS frame; (**iv**) the gelatin microfluidic device.

The casting process used to fabricate the crosslinked gelatin device is shown in Figure 1. The outer frame was placed on the PDMS master mold [Figure 1(A,Biii)]. To create inlet and outlet ports, polytetrafluoroethylene (PTFE) tubes (1 × 2 × 6 mm; Nichias, Tokyo, Japan) covered with Tygon tubes (1.59 × 3.18 × 2 mm; Saint-Gobain, Courbevoie, France) were placed on both ends of the microchannel pattern. The uncured gelatin mixture (1 mL) was poured over the PDMS master mold and incubated at 37 °C for 5 h to crosslink the gelatin, followed by incubation at 4 °C before the bonding process.

The crosslinked gelatin device was removed from the PDMS mold and outer frame. Excess moisture was wiped from the bonding surface of the gelatin device, and then the device was bonded with an APTES- GA-modified glass coverslip at 4 °C for at least 1 h. After bonding, the gelatin device was transferred to a plastic box and then heat-treated at 65 °C in PBS (−) for 1 h to inactivate and remove the enzyme. Next, the gelatin device was transferred to a 6-well cell culture plate under sterile conditions. Culture media appropriate for each type of cell (5 mL) were added to the wells, and the device was incubated at 37 °C with 5% CO_2_ overnight.

### 2.4. Fabrication of PDMS Microfluidic Devices

The PDMS device was fabricated as previously described [5,7,8]. Through-holes were made at both ends of the microchannel in a PDMS sheet using a 2.0-mm biopsy punch (BP-20F; Kai Industries, Tokyo, Japan). Both the bonding surfaces of the PDMS sheet and coverslip (24 × 24 mm) were exposed to oxygen plasma at 100 W for 35 s. The PDMS sheet was bound to the coverslip and baked at 100 °C for 1 h. Each hole was connected to a PTFE tube (inner diameter, 1 mm; outer diameter, 2 mm; length, 7 or 8 mm; Nichias). The PTFE tube was glued to the PDMS sheet with the PDMS prepolymer and baked at 100 °C for 1 h. The devices were sterilized by autoclaving.

### 2.5. Fabrication of Stripe-Micropatterned Gelatin Wells

**Figure 2:**
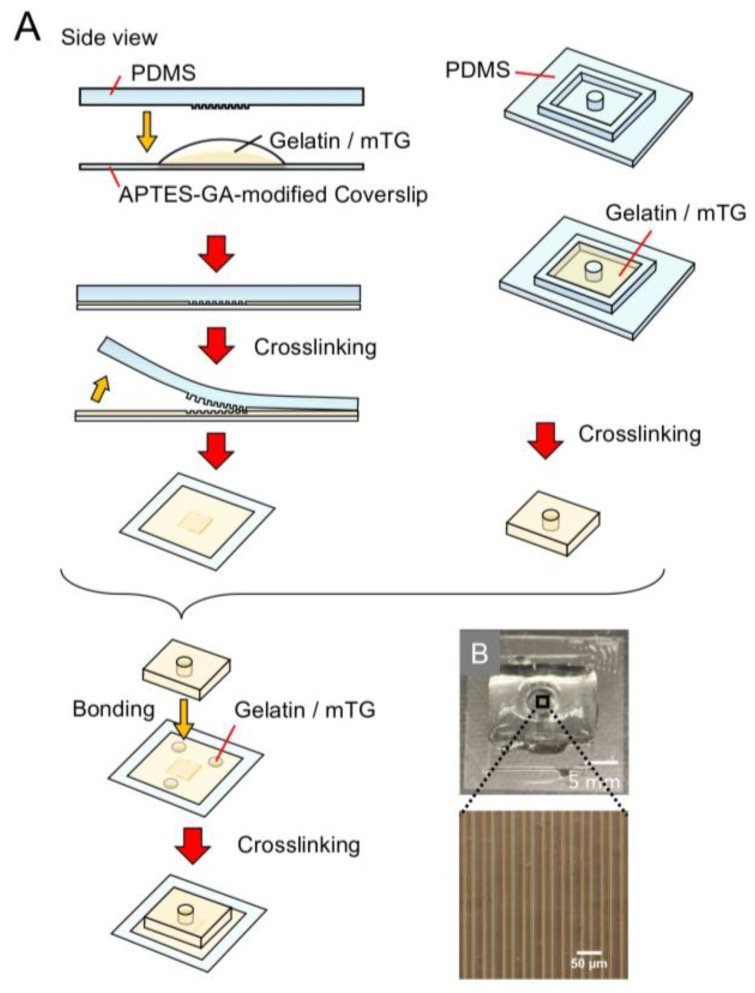
Fabrication process of the stripe-patterned gelatin gel on a coverslip in a gelatin well: (**A**) schematic illustration of the fabrication process; (**B**) photograph of a stripe-patterned gelatin gel in a gelatin well.

The fabrication of the striped micropatterned gelatin well is shown in Figure 2. The patterning of the stripe-line structure on the gelatin sheet was performed as reported by Mccain et al. [14,15,18,19] and our research group [46]. The SU-8 3005 molds were fabricated by photolithography using a chrome mask [47] with a line width and spacing of 20 μm. The PDMS stamps were replicated from the SU-8 3005 mold. The crosslinked gelatin gel was stripe-micropatterned on the APTES-GA-modified glass coverslip by pressing with a PDMS stamp with ridges/grooves of 20/20 μm in width and 1, 6, or 10 μm in depth. The uncured gelatin mixture (100 µL) was poured over an APTES-GA-modified glass coverslip. Next, a degassed PDMS stamp was placed on the uncured gelatin mixture immediately, and the gelatin gel was incubated for 5 h at 37 °C and then at 4 °C before the bonding process. After polymerization, the coverslip with stripe-micropatterned gelatin was removed from the mold just before bonding with a 5 mm thick crosslinked gelatin sheet with a 5 mm diameter hole using an uncured gelatin and mTG mixture as a glue. A small amount of the gelatin and mTG mixture was placed on the coverslip, and the gelatin sheet was placed on the coverslip, ensuring that the micropattern was at the center of the 5 mm diameter hole. The device was then incubated at 37 °C for 5 h. After bonding, the gelatin device was transferred to a plastic box and then heat-treated at 65 °C in PBS (−) for 1 h to inactivate and remove the enzyme. Next, the gelatin device was transferred to a 6-well cell culture plate under sterile conditions. Cell culture media appropriate for each type of cell (5 mL) were added to the wells, and the device was incubated at 37 °C with 5% CO_2_ overnight.

### 2.6. Gel Indentation Assay

A fluorescent bead suspension (20 µL, Fluoresbrite YG Microdphres-1.00 µm; Polysciences, Warrington, PA, USA) was dropped onto the surface of a 1 mm thick gelatin gel substrate and allowed to stand for 1 min. To remove excess fluorescent beads that were not attached to the gel surface, the surface was washed three times with PBS (−). The gel was set on the microscope, and the *z*-axis position was measured where the fluorescent beads were in focus. A stainless-steel sphere (0.25 mm) was then placed on the gel surface, and the *z*-axis position was measured again after the stainless-steel sphere sank and stopped in the gel. The difference between the two *z*-axis positions was calculated as the indentation depth, and the results of the three measurements were averaged.

The formula for calculating the elastic modulus *E* (Pa) is as follows [48]: the density of the stainless-steel sphere was 7.93 × 10^3^ kg/m^3^, and Poisson’s ratio was 0.45 [48], which corresponds to the general soft-tissue organs.

$$E=\frac{3\left(1-v^{2}\right)F}{4R^{1/2}\delta^{3/2}}$$

*E* = Elastic modulus, Pa.

*ν* = Poisson’s ratio.

*F* = weight of ball (volume × density), N.

*R* = elastic modulus ball radius, m.

*δ* = experimental height, m.

### 2.7. Scanning Electron Microscopy (SEM) Imaging of Gelatin and PDMS Surface

Gelatin, PDMS, and glass substrates were prepared for observation by SEM. Gelatin substrate was prepared as follows: the APTES-GA-modified coverslips were overlaid with a PDMS strip with a hole of 8 mm in diameter to form a well structure, and 100 µL of gelatin solution containing mTG was poured into the well. The gelatin was crosslinked at 37 °C for 5 h and inactivated at 65 °C for 30 min. For PDMS and APTES-GA-modified coverslips, a well structure was made with a PDMS sheet with 8 mm diameter holes, as for gelatin substrates.

For surface coating with fibronectin, 60 µL of 0.1 mg/mL fibronectin was added to the wells and incubated overnight. Fibronectin was then carefully removed using a pipette. The substrates were washed twice with 100 µL of EGM 2, fixed for over 4 h with 5 mL of 2.5% GA in 1/15 M PBS (pH 7.4), and then washed three times with 1/15 M PBS for 10 min each. Subsequently, the samples were incubated for 10 min with 0.5% OsO_4_ in 1/15 M PBS and washed three times with deionized water for 5 min each. Dehydration steps were performed three times with 30, 50, 70, 80, 90, and 95% ethanol for 10 min each, and then in 100% ethanol for 30 min. The samples were kept in 100% ethanol for 2 d at 4 °C and then dried using the critical point dryer (Leica EM CPD300, Leica Microsystems, Wetzlar, Germany). Finally, the samples were mounted on stubs and sputter-coated with gold-palladium using an ion sputter coater (E-1030; Hitachi High-Tech, Tokyo, Japan). The specimens were observed and photographed using a field-emission SEM (SU8220, Hitachi High-Tech) at an accelerating voltage of 2.0 kV.

### 2.8. Microfluidic Cell Culture

Normal human dermal fibroblasts (NHDF), neonatal (NHDF-Neo, Lot.0000251354; Lonza, Basel, Switzerland), and human umbilical vein endothelial cells (HUVEC, (Lot.405Z013; PromoCell, Heidelberg, Germany) were used. NHDFs were grown in fibroblast growth Medium-2 (FGM 2, PromoCell), and HUVECs were grown in endothelial cell growth Medium-2 (EGM 2, PromoCell). An antibiotic-antimycotic solution (Thermo Fisher Scientific, Waltham, MA, USA) was added to both cell culture media. NHDFs before the eighth passage and HUVECs before the ninth passage were used in all experiments. Once the cells reached 80% confluence in a cell culture flask, the medium was aspirated, and the cells were rinsed with 5 mL of PBS (−) (Takara Bio Inc., Shiga, Japan) and treated with 1 mL of TrypLE Express (Thermo Fisher Scientific). After the cells were detached from the surface of the flask, 2 mL of fresh medium was added, and the obtained cell suspension was transferred to a 15-mL conical tube. The tube was centrifuged at 1200 rpm for 5 min, the supernatant was aspirated, and the cells were resuspended in the culture medium at the required concentration.

Before introducing the cell suspension, the PDMS microchannel was coated with fibronectin by incubating it with 0.1 mg/mL fibronectin (from Human Plasma, FUJIFILM Wako Pure Chemical) at 37 °C for 1 h, whereas the gelatin microchannel was not coated with fibronectin. For the monolayer culture, the NHDF suspension was prepared at 5 × 10^6^ or 1 × 10^7^ cells/mL, and 20 µL of the culture was added to the 100-µm-width microchannel. The channels were incubated for a minimum of 1 h at 37 °C to allow the cells to attach to the bottom of the channel. Another 20 µL of the cell suspension was added again to the channel, and the device was rotated 180° and incubated for 1 h at 37 °C. This process was repeated with subsequent 90° or 180° rotations until all four sides of the channel walls were coated. For the 200-µm-wide microchannel, 50–100 µL of a 4 × 10^6^ cells/mL suspension was used. Other cell-seeding conditions are described below. For the NHDF and HUVEC co-culture, HUVECs were seeded onto the NHDFs attached to the microchannel walls. After cell attachment, the device was incubated at 37 °C for a minimum of 12 h. The PDMS device was wrapped with a wet, lint-free wiper (BEMCOT M-1; Asahi Kasei, Tokyo, Japan) to prevent desiccation. The gelatin device was incubated in a 6-well cell culture plate filled with medium.

### 2.9. Microfluidic Perfusion

After HUVECs were cultured as a monolayer on the top and bottom surfaces of the gelatin channel (width, 200 µm) for 18 h, the medium was infused into the channel using a syringe pump (Model Fusion 720; Chemyx, Stafford, TX, USA). For fluidic culture, the PTFE tube from the inlet of the channel was connected to a 5-mL syringe (Terumo, Tokyo, Japan) via a bubble trap [3,4,5,7,8], a PFA capillary (0.3 × 0.5 × 900 mm), and a 19-G needle (Nonaka Rikaki, Tokyo, Japan). The other PTFE tube from the outlet of the channel was connected to a Tygon tube (1.59 × 3.18 × 100 mm). The flow rate was increased stepwise every 10 min from 0.3 mm/s to 13 mm/s over 110 min.

### 2.10. Co-Culture on Stripe-Micropatterned Surfaces

HUVECs (passage number 6, 2.2 × 10^5^ cells/mL, 20 µL) were seeded in the gelatin well with a stripe-micropatterned gelatin surface and cultured with EGM 2 for 24 h. Next, NHDFs (passage number 8, 8.8 × 10^6^ cells/mL, 20 µL) were seeded on the HUVECs in the gelatin well and cultured with FGM 2 for 8 d to construct the capillary network of HUVECs. During this process, the medium was exchanged six times.

### 2.11. Cell Viability Assay

Cell viability assays were performed using two fluorescent dyes. Cells were incubated with 2 μM calcein AM (Dojindo, Kumamoto, Japan) and 4 μM ethidium homodimer (FUJIFILM Wako Pure Chemical) in the culture media for 30 min at 37 °C and 5% CO_2_, and then rinsed with PBS (+) twice.

Fluorescence images were obtained using an IX83 microscope (Olympus) equipped with a 100-W high-pressure mercury lamp and a cooled CCD camera, ORCA-R2 (Hamamatsu Photonics, Hamamatsu, Japan). A dichroic mirror block U-FGW (excitation 530–550 nm, emission > 575 nm) was used to observe the ethidium homodimer. To observe calcein AM, another dichroic mirror block, U-FBNA (excitation 470–495 nm, emission 510–550 nm), was used. Phase-contrast images were obtained using a CKX53 microscope (Olympus).

### 2.12. Cell Staining

To stain F-actin, cell nuclei, CD34, VE-cadherin, and fibronectin, cells were washed three times with 50–100 µL of PBS (+)for 1 min each, fixed with 50—100 µL of 4% paraformaldehyde (PFA; Alfa Aesar, Tewksbury, MA, USA) at 4 °C for 15–20 min, and then permeabilized with 0.1% Triton X-100 in PBS (+) for 15 min at 23 °C.

To stain F-actin, fixed cells in the gelatin device were reacted with 50 µL of 0.33 µm rhodamine phalloidin (Thermo Fisher Scientific) or 0.28 μm phalloidin, green fluorescent conjugate, acti-stain 488 (Cytoskeleton, Denver, CO, USA) in 0.1% Triton X-100 in PBS (+) for 30 min at 23 °C. For cells in the PDMS device, 0.5% bovine serum albumin (BSA) or an equal volume mixture of 2% BSA and 5% goat serum (Thermo Fisher Scientific) in PBS (+) was used as a dilution buffer instead of 0.1% Triton X-100 in PBS (+), and cells were incubated for 16 h at 4 °C. After staining F-actin, the cells were rinsed twice with PBS (+) for 1 min each time. Cell nuclei were stained with 10 μg/mL Hoechst 33342 (Thermo Fisher Scientific) in Milli-Q water for 10 min at 23 °C and then rinsed with PBS (+).

Primary and secondary antibodies were used, as previously described. To stain CD34, 0.6 μg/mL mouse anti-human CD34 class II clone QBEnd 10 monoclonal (Dako M7165; Agilent, Santa Clara, CA, USA) and 4 μg/mL Alexa Fluor Plus 555 goat anti-mouse IgG (H + L) antibodies (A32727; Thermo Fisher Scientific) were used. For VE-cadherin/CD144 staining, 10 μg/mL rabbit anti-human VE-cadherin monoclonal IgG (ab33168; Abcam, Cambridge, UK) and 4 μg/mL Alexa Fluor Plus 555 goat anti-rabbit IgG (H + L) (A32732; Thermo Fisher Scientific) antibodies were used. For fibronectin staining, 10 μg/mL anti-fibronectin mouse monoclonal IgG (SC-8422; Santa Cruz Biotechnology, Dallas, TX, USA) and 10 μg/mL Alexa Fluor Plus 647 goat anti-mouse IgG (H + L) antibodies (A32728; Thermo Fisher Scientific) were used.

To stain CD34, VE-cadherin, and fibronectin, cells were fixed and treated with Triton X-100 and blocked with PBS (+) containing 0.5% BSA (FUJIFILM Wako Pure Chemical), an equal volume mixture of 2% BSA and 5% goat serum, or Blocking One-P (05999; Nacalai Tesque, Kyoto, Japan) for 30–60 min at 23 °C. Next, cells were incubated with the primary antibody in the blocking buffer for 16 h at 4 °C, rinsed with PBS (+) twice for 2 min each, and reacted with the secondary antibody in the blocking buffer for 30–60 min at 23 °C, and rinsed with PBS (+) twice for 2 min each. Stained cells were fixed again with 50–100 µL of 4% PFA for 5 min at 23 °C and rinsed with PBS (+) twice for 2 min each. All images were obtained using a confocal laser-scanning microscope (FluoView FV1200, Olympus). To observe the stained cells, 405 nm, 473 nm, 559 nm, and 635 nm LD lasers were used. Conditions were as follows; Laser power 30%, HV 535 V, Gain 1×, Offset 6% for 405 nm; Laser power 30%, HV 650 V, Gain 1×, Offset 6% for 473 nm; Laser power 10%, HV 460 V, Gain 1×, Offset 6% for 559 nm; Laser power 15%, HV 460 V, Gain 1×, Offset 6% for 635 nm. Data analysis of fluorescence images (8-bit) was performed using Image J (National Institutes of Health, Bethesda, MD, USA).

## 3. Results and Discussion

### 3.1. Characterization of Gelatin-Based Devices

The elastic modulus of the gelatin sheets was measured (Figure S1) [48]. The indentation depths of 10 wt% gelatin gels crosslinked with 1 U/mL mTG for 5 h at 37 °C measured by the radius of 0.25 mm stainless-steel ball at three locations were 1.6, 2.0, and 1.4 μm, respectively. The gel indentation assay indicated that the stiffness of the gelatin gel was 93.0 ± 24.1 kPa (*n* = 3), which is similar to a previous report [19]. This value is similar to the stiffness of muscles [49]. The modulus of PDMS has been reported to be 580 kPa [50], which is comparable to that of cartilage [51] and higher than that of our gelatin gel. The modulus of glass coverslip has been reported to be approximately 80 GPa [52], and thus, it is much stiffer than biological tissue [51]. Therefore, gelatin gel is better suited than PDMS or glass for mimicking soft tissues (<100 kPa). Moreover, decreasing the mTG concentration resulted in softer sheets, whereas a 0.1 U/mL mTG concentration resulted in stickier sheets (Figure S2). These results indicate that 1 U/mL mTG is suitable for a gelatin gel mold for the fabrication of a microchannel structure.

Gelatin gels with 1 U/mL mTG allowed the fabrication of a microchannel with a width and height of over 100 µm. Photographs of the SU-8 master mold (concave structure), the PDMS mold (convex structure) made by molding the SU-8 master, and the gelatin device with the microchannel made by molding the PDMS mold and sealed with a glass coverslip are shown in Figure 1(Bi–iv). In the PDMS device, a tube connection can be performed by the simple insertion of plastic tubing into the hole at each end of the channel. However, in the gelatin device, this method causes the tube to exit the hole (Figure S3). However, by using a PTFE tube (i.d., 1 mm; o.d., 2 mm; length, 6 mm) covered with a short Tygon tube (i.d., 1.59 mm; o.d., 3.18 mm; length, 2 mm) as a stopper, the tube was successfully fixed to the gelatin sheet, allowing for stable liquid introduction (Figure S3). Therefore, we were able to create durable gelatin-based devices that were comparable to PDMS-based ones.

Figure 2B shows an overview of the gelatin well with a striped structure on the coverslip. The measured line, gap width, and height of the PDMS mold were 17.9 ± 0.6, 19.4 ± 0.8, and 15.7 ± 0.2 µm, respectively (*n* = 5). Using this PDMS mold as a template, the measured line, gap widths, and height of the gelatin stripes were 11.3 ± 0.9, 16.8 ± 2.7, and 9.7 ± 1.4 µm, respectively (*n* = 5). The dimensions of the gelatin structure were smaller than those of the PDMS mold due to the shrinkage of the gelatin gel; however, the height of the stripe can be changed by altering the thickness of SU-8.

Next, we analyzed the permeability of the gelatin gel from the channel to the channel wall using 10 µm FITC-dextran (FW = 40,000) as a high-molecular-weight fluorescent tracer, and 10 µm fluorescein (FW = 376) as a small-molecule tracer and tracers dissolved in the culture media were introduced into the gelatin channel (1 × 1 × 10 mm) without cells. The fluorescence intensity of dextran diffused into the walls of the gelatin channels was measured for 60 min. The intensity distributions were obtained by line-scanning the fluorescent images near the center of the channels using the plot profile function of the ImageJ software. Our results revealed that the gelatin wall exhibited a higher diffusion rate of fluorescein than that of dextran (Figure S4). Moreover, the intensity of fluorescein in the gelatin wall continuously increased with time, whereas that of dextran only slightly increased. These results indicate that gelatin-based devices can provide a cell culture environment that prevents the accumulation of small metabolic waste molecules.

### 3.2. Monolayer Cell Culture in the Gelatin Channel

**Figure 3:**
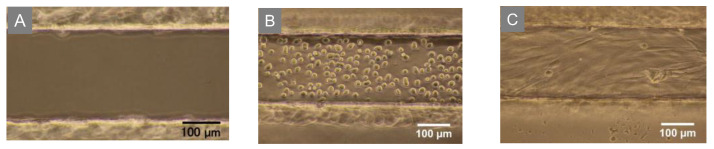
Photograph of the gelatin channel (200 μm width); (**A**) empty channel; NHDF cultured on (**B**) day 0 and (**C**) day 1.

**Figure 4:**
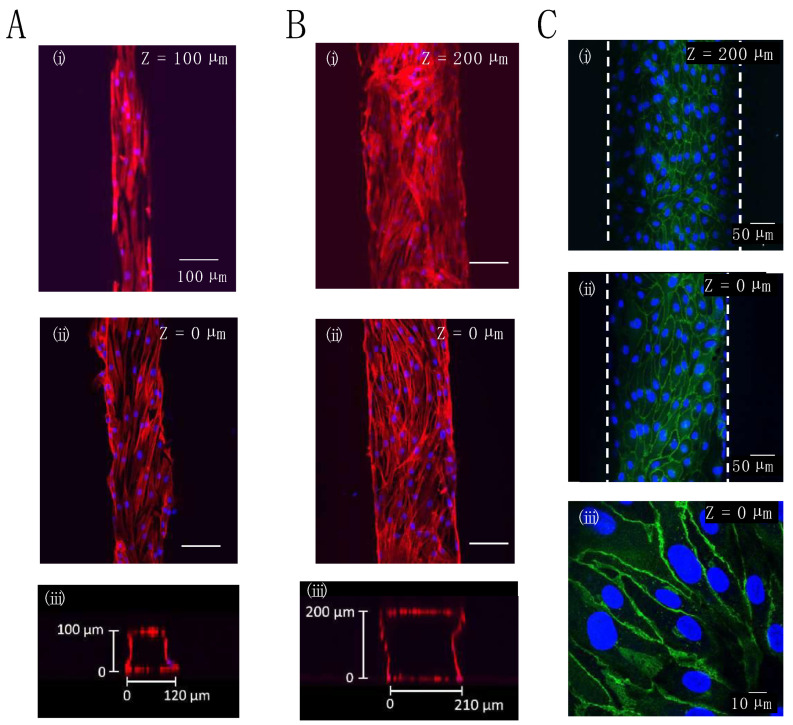
Confocal microscopy images of stained cells present inside the microchannel of the gelatin device: (**A**,**B**) F-actin (red) and nuclei (blue) staining of NHDFs adhered at the top (**i**) or bottom (**ii**) of a microchannel, and confocal microscopy image of the cross-section of a microchannel (**iii**), the difference between (**A**,**B**) is the channel width and height; all other conditions were the same; (**C**) VE-cadherin (green) and nuclei (blue) staining of HUVECs adhered at the top (**i**) or bottom (**ii**,**iii**) of a microchannel. The dimensions of the microchannel were 100 μm width × 10 mm length × 100 μm height for (**A**) or 200 μm width × 10 mm length × 200 μm height for (**B**,**C**).

Because the elasticity of gelatin gel is not comparable to that of PDMS, the inlet tubing tends to pull out easily. However, in the device constructed herein, the procedures, including cell introduction and medium change, were successfully performed without any special care, similar to culture in a PDMS device. We confirmed that the cells could be seeded and cultured without any problems, such as inlet tubing disconnection (Figure 3). Our gelatin device features the use of a coverslip at the bottom of the microchannel, which allowed us to observe cultured cells by confocal microscopy. Previous reports [28,29,30,32] did not use a coverslip at the bottom of the microchannel. High-magnification objective lenses are difficult to use when a thick gelatin sheet is used on the bottom. Figure 4 shows confocal images of the monolayer cell culture in gelatin microchannels (0.1 × 0.1 × 10 mm or 0.2 × 0.2 × 10 mm). NHDFs were introduced into the channels and adhered to the four sides of the inner wall (Figure 4A,B). Similarly, HUVECs adhered to the four inner walls of the gelatin channel with a width and depth of approximately 200 µm. Further, HUVECs formed a confluent monolayer with tight cell-cell junctions at both the top of the gelatin channel (gelatin surface) and the bottom coverslip after three days of cell culture, as revealed by VE-cadherin (a vascular endothelial cell-cell adhesion glycoprotein) staining (Figure 4C). These findings suggest that a favorable culture environment could be maintained in a narrow 100 µm wide and deep gelatin channel under static conditions, a feat that is difficult to achieve with PDMS devices [7].

### 3.3. Observation of Fibronectin by Confocal Microscopy and SEM

**Figure 5:**
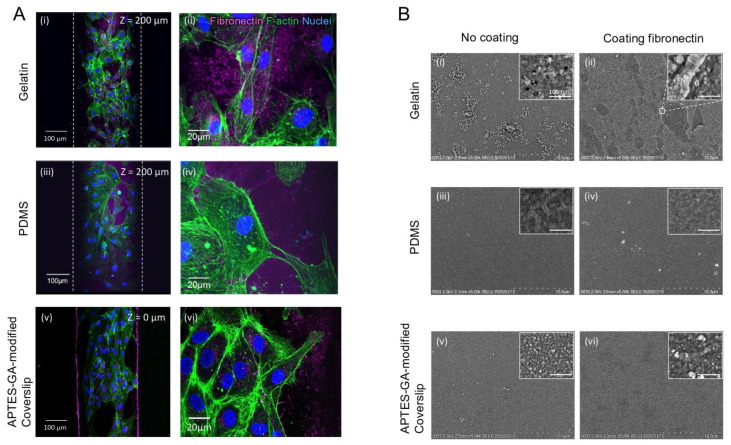
Confocal microscopy and scanning electron microscopy images (SEM): (**A**) Confocal microscopy images of HUVEC labeled for fibronectin (purple), F-actin (green), and nuclei (blue) on the gelatin (**i**,**ii**), PDMS (**iii**,**iv**), or APTES-GA-modified glass coverslips (**v**,**vi**); (**B**) SEM images of the gelatin (G) (**i**,**ii**), PDMS (**iii**,**iv**), or APTES-GA-modified glass coverslip (**v**,**vi**) with or without fibronectin (FN).

We investigated the distribution of fibronectin on gelatin, PDMS, and APTES-GA-modified glass surfaces in the microchannel using confocal microscopy and SEM. First, we observed HUVECs and fibronectin attached to the top and bottom of gelatin- or PDMS-glass hybrid microchannels using confocal microscopy. Fibronectin fibers were strongly detected on the gelatin surface [Figure 5(Ai,ii); mean fluorescence intensity (MFI) was 30 ± 23; maximum (Max) and minimum (Min) intensities were 159 and 2, respectively. Fibronectin fluorescence intensity distribution maps are shown in Figure S5]. In contrast, thin film-like fibronectin was detected on the PDMS surface [Figure 5(Aiii,iv); MFI, Max, and Min were 30 ± 15, 58, and 15, respectively]. Although fibronectin fibers were detected on the surface of APTES-GA-modified glass [Figure 5(Av,vi); MFI, Max, and Min were 14 ± 16, 171, and 1, respectively], they were more strongly detected on the gelatin surface. In addition, fibronectin attaches to the PDMS surface via physical adsorption, whereas it specifically attaches to gelatin [53], which may result in stronger interactions in gelatin-based devices than PDMS-based ones.

Next, the surfaces of these three types of substrates, with or without fibronectin, were observed using SEM (Figure 5B). HUVECs were not used in this experiment as we were only interested in the binding of fibronectin to the substrate. The surface of the bare gelatin gel was rough [Figure 5(Bi)], and small pores (diameter < 15 nm) were observed under 100,000× magnification [Figure 5(Bi), inset]. These small pores can allow the entry of molecular-sized substances such as salts, sugars, amino acids, and small proteins. The gelatin surface with fibronectin showed non-uniform film-like fibronectin [Figure 5(Bii)], and the magnified view in the inset shows the edge of the film-like fibronectin. Small pores similar to those observed in the bare gelatin gel were observed only in the area where fibronectin peeled off from the gelatin surface. The PDMS surface was smooth [Figure 5(Biii)]. Fibronectin was not observed on the PDMS substrate [Figure 5(Biv)]. Fibronectin may have been stripped off during dehydration with ethanol. The APTES-GA-modified glass surface was smooth [Figure 5(Bv)]. A layer of fibronectin was observed on the APTES-GA-modified glass surface [Figure 5(Bvi)], which was thinner than that observed on the gelatin surface. These observations suggest that the affinity of fibronectin for gelatin is stronger than that for glass and PDMS and that gelatin is a suitable substrate for cell adhesion.

### 3.4. Microfluidic Culture of HUVECs

**Figure 6:**
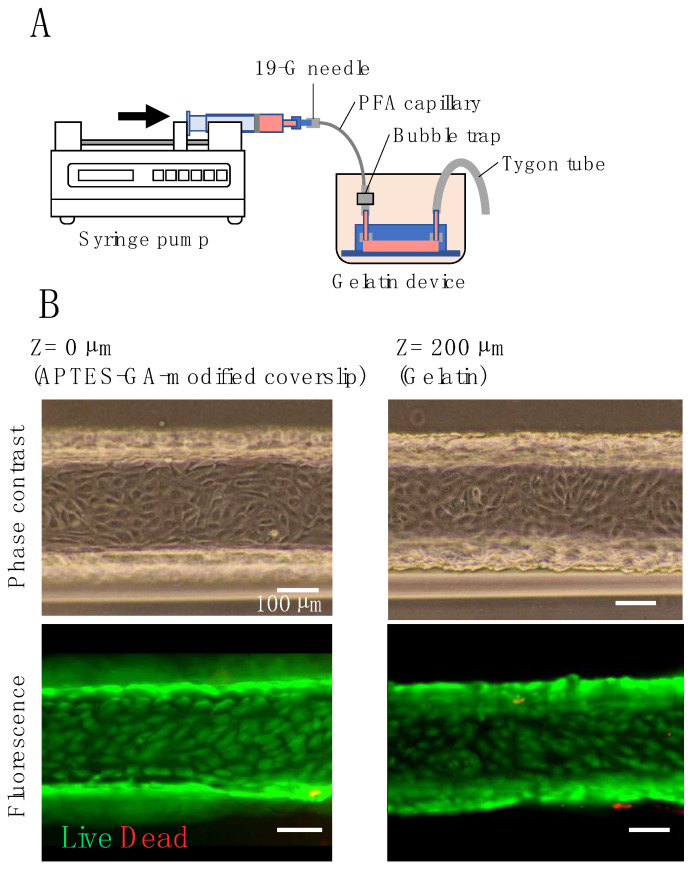
Perfusion cell culture: (**A**) illustration of the gelatin device with a syringe pump for perfusion culture; (**B**) phase-contrast and live/dead assay images of HUVECs in a gelatin channel before and after perfusion.

In our gelatin-based device, a gelatin microchannel was used for the fluidic cell culture. This microchannel must be cultured under conditions of linear velocity equivalent to that of blood flow in capillaries (0.3 mm/s), arterioles (6 mm/s), and small arteries (13 mm/s) in order to be used as a model for blood vessels [54]. Figure 6A shows the setup of the fluidic culture. To avoid drying, the device was placed in a cup containing a culture medium. A syringe pump was used to pump the medium through the flow channel. There was no leakage of the medium from the flow channel or the gap between the inlet tubes during the flow culture. Phase-contrast images (Figure 6B) show HUVECs at both the top of the gelatin channel (gelatin surface) and the bottom coverslip 16 h after cell seeding. The cells were confluent (8.7 × 10^4^ cells/cm^2^ for APTES-GA-modified cover slip and 9.0 × 10^4^ cells/cm^2^ for gelatin) and in close contact with each other. Fluorescent images (Figure 6B) show the results of the live-dead assay after fluidic culture, in which the flow rate was increased stepwise every 10 min from 0.3 mm/s to 13 mm/s over 110 min. There was no cell detachment at either the top or bottom of the channel, indicating that most HUVECs were attached as viable cells (green). The cell densities on APTES-GA-modified coverslip and gelatin after perfusion were 8.0 × 10^4^ and 8.6 × 10^4^ cells/cm^2^, respectively, which were 90% or more of the density before perfusion. These results showed that HUVECs cultured in our gelatin device withstood flow stimuli equivalent to the linear velocity of small arteries (13 mm/s) [54].

### 3.5. Co-Culture of HUVECs and NHDFs in the Gelatin Channel

**Figure 7:**
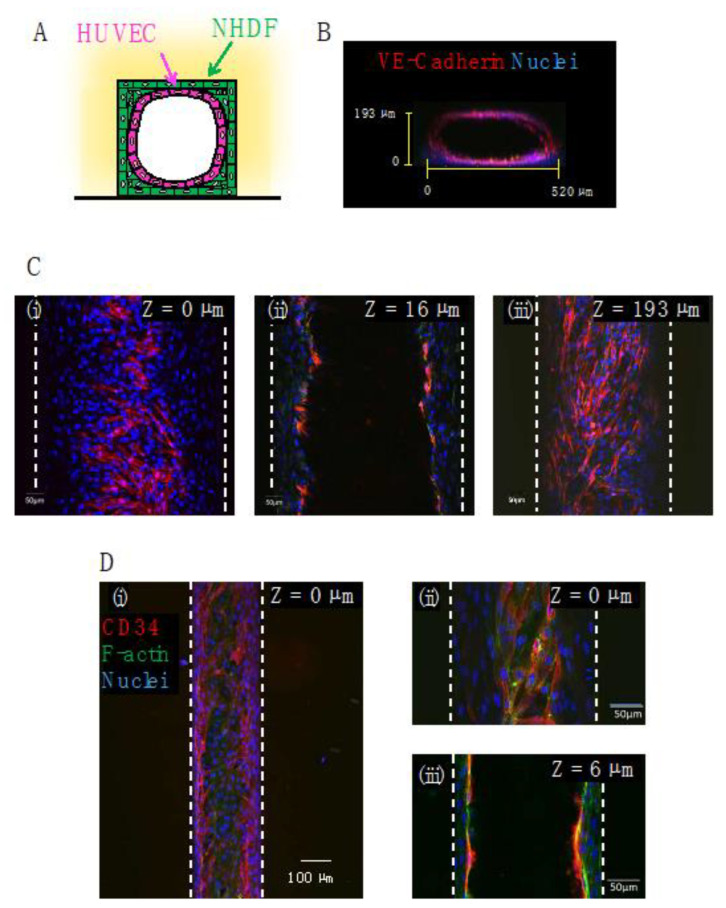
Confocal microscopy images of HUVECs and NHDFs co-cultured in gelatin microchannel devices: (**A**) schematic illustration of the co-culture model. HUVECs were seeded onto multilayer NHDFs; (**B**) confocal microscopy image of the cross-section of a microchannel. The dimensions of the microchannel were 540 μm width × 17 mm length × 200 μm height. VE-cadherin (red) and nuclei (blue); (**C**) confocal microscopy images of the same microchannel as for (**B**) at three different heights, (**i**) z = 0 µm (cells adhered at the bottom of a microchannel), (**ii**) 16 µm (cells adhered at the side wall of a microchannel) and (**iii**) 193 µm (cells adhered at the top of a microchannel) from the bottom of the channel, respectively; (**D**) confocal microscopy images at the bottom [(**i**,**ii**) z = 0 µm] and side wall [(**iii**) z = 6 µm] of a microchannel of 200 μm width × 10 mm length × 200 μm height. CD34 (red), F-actin (green), and nuclei (blue).

HUVECs were co-cultured on the surface of the NHDFs in a gelatin channel (Figure 7). Figure 7B shows a cross-sectional fluorescence image of a gelatin channel (w × h × l = 0.54 × 0.2 × 10 mm) after 1 day of co-culture of monolayer HUVECs and multilayer NHDFs. Although the cross-section of the channel was rectangular, the HUVEC layer overlapping the NHDFs was cylindrical. Figure 7B,C show fluorescence images of a co-culture of HUVECs and NHDFs stained with anti-VE-cadherin and Hoechst33342 (nuclei) in the gelatin channel (w × h × l = 0.54 × 0.2 × 10 mm). HUVECs formed tight cell-cell junctions at the top (z = 193 µm) and bottom (z = 0 µm) of the gelatin channel. Figure 7D shows a fluorescence image of the co-culture of HUVECs and NHDFs stained with anti-CD34 (endothelial marker) and Hoechst33342 in the gelatin channel (w × h × l = 0.2 × 0.2 × 10 mm). The z = 16 µm photograph in Figure 7C and the z = 6 µm photograph in Figure 7D show that the inner wall of the microchannel was covered with a multilayer of NHDFs and a monolayer of HUVECs, resulting in a structure that mimicked vascular tissue. Previous reports of gelatin devices typically involved monolayer cultures [28,29,30,31,32,33,35,36]; however, this experiment demonstrated co-cultures in a gelatin microchannel that mimics blood vessels.

### 3.6. Formation of Capillary/Vessel-like Structure in the Gelatin Microstructure

**Figure 8:**
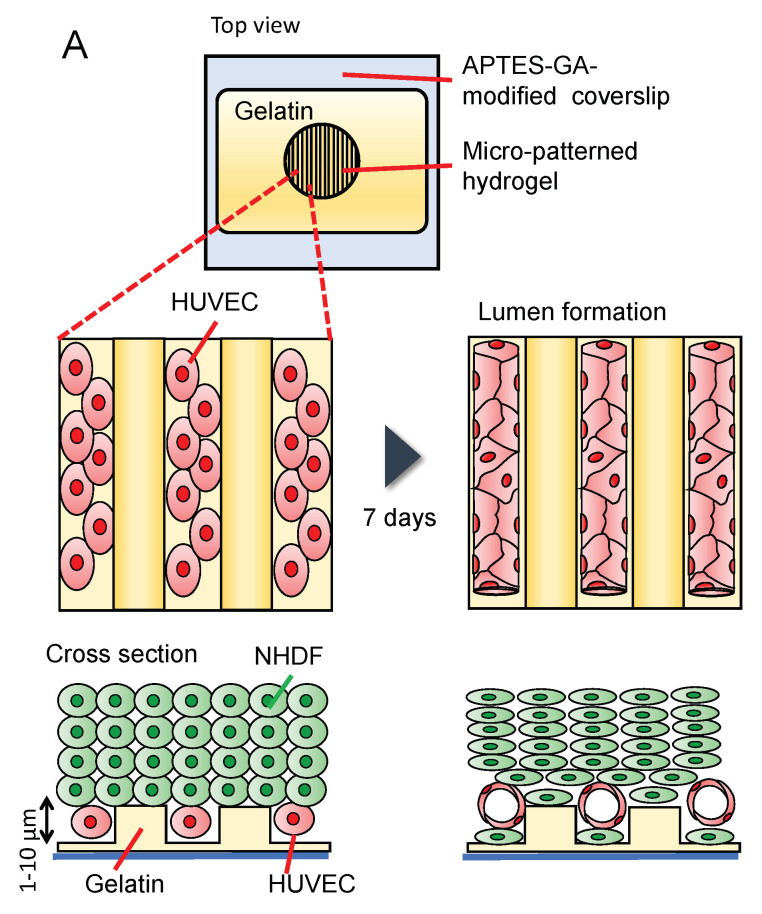

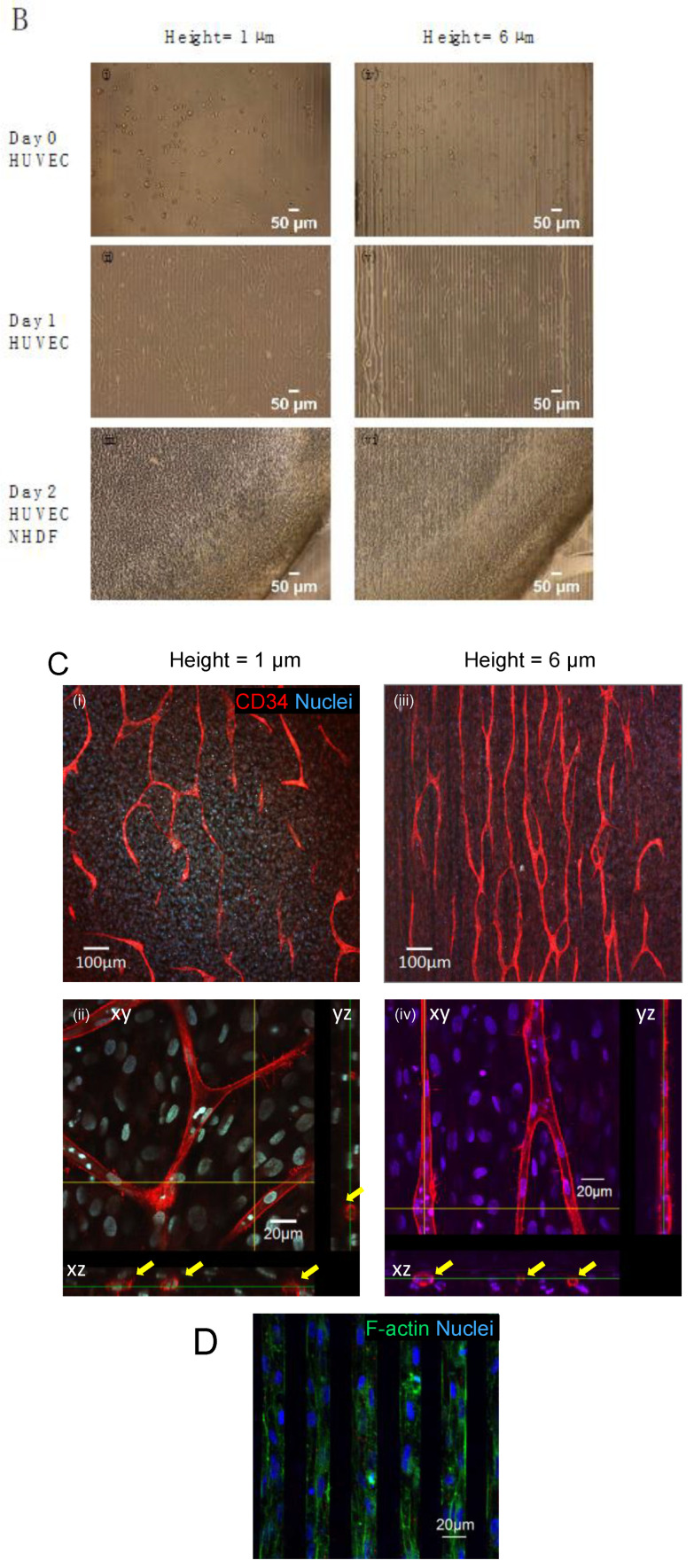
HUVECs co-cultured with NHDFs in gelatin wells forms a capillary-like structure: (**A**) Schematic illustration of the co-culture model. HUVECs are first seeded on the gelatin pattern, and then NHDFs are seeded on HUVECs; (**B**) phase-contrast images of HUVECs and NHDFs on stripe gelatin gel with a height of 1 µm (**i**–**iii**) or 6 µm (**iv**–**vi**) and 20 µm-groove widths; (**C**) confocal microscopy images of the HUVEC capillary-like network (red) eight days after co-culture with NHDFs (unlabeled) on stripe gelatin gel with a height of 1 µm (**i**,**ii**) or 6 µm (**iii**,**iv**) and 20 µm groove width; (**D**) confocal microscopy image of NHDF (green) residing inside the grooves (1 µm height).

The diameter of an in vivo capillary lumen is approximately 6 µm [54]; however, it is difficult to introduce cells into gelatin microchannels less than 100 μm in width. Therefore, we investigated the spontaneous formation of a three-dimensional vascular network in HUVECs using an open gelatin device. Wells with a gelatin stripe structure on a coverslip were used for the on-chip vasculature formation. Stripe structures of gelatin with different heights (1 or 6 µm) were used for culturing. HUVECs were seeded on the gelatin stripe structure, and the next day, NHDFs were seeded on the HUVEC layer to form multiple layers using the cell accumulation technique [55], and the cells were cultured for seven days (Figure 8A). Figure 8B shows phase-contrast images of the cells. Immediately after seeding (day 0), HUVECs had a round shape. On day 1, regardless of the orientation of the gelatin stripe with a height of 1 µm, the cells spread in random directions [Figure 8(Bii)]. In contrast, the cell orientation was aligned along the gelatin stripe at the height of 6 µm [Figure 8(Bv)]. On day 2, multiple layers of NHDFs were observed on top of HUVECs [Figure 8(Biii–vi)]. Confocal microscopy images after eight days of culture are shown in Figure 8C. A parallel capillary-like structure was observed, particularly with stripes with a height of 6 µm [Figure 8(Ciii,iv), Video S1]. HUVECs were likely oriented along the striped substrate with a width of 20 µm. Nishiguchi et al. coated the cell surface with gelatin and fibronectin before culturing the cells [55]; however, they observed the formation of three-dimensional multilayered tissues without this step. In our study, in the confocal reconstruction cross-sectional view images, we observed the formation of a lumen by HUVECs, indicating that tube-like structures with a diameter of approximately 10 µm were successfully constructed [Figure 8(Cii–iv), yellow arrow]. Figure 8D shows NHDFs attached to the gelatin stripe on the coverslip. Figure 8D only shows cells seeded at a stripe with a height of 1 µm; however, similar results were obtained with stripes with a height of 6 µm. Although HUVECs were placed on the bottom surface immediately after cell seeding, NHDFs moved to the bottom, and blood vessels formed above the NHDF layer as the HUVECs began to form a capillary-like structure.

In addition, no differences in the PDMS and gelatin structures were observed between the lumen devices when comparing the striped structures and wells made of gelatin to those made of PDMS (Figure S6A). This indicates that a vascular structure was constructed along the striped structure, regardless of the material. However, in the PDMS devices, cell detachment from the walls was observed, concomitant with cell shrinkage (Figure S6B), whereas cells remained adherent to the walls in our gelatin device. If the tension between cells becomes stronger in long-term culture, it will probably exceed the binding force between PDMS and fibronectin, causing fibronectin to detach from the PDMS surface and the cells bound to fibronectin to detach from the PDMS surface. Taken together, these results suggest that there is an advantage to using gelatin wells over PDMS wells if cell detachment is to be prevented.

## 4. Conclusions

In this study, we produced a cell culture device using gelatin and a coverslip. The device is transparent and suitable for microscopic observation. The PTFE tubing placed in the inlet hole was designed to be difficult to dislodge, and it was possible to flow liquid at a linear velocity equivalent to that of the blood flow. HUVECs and NHDFs were successfully co-cultured using this device. Moreover, we observed better adsorption of fibronectin on gelatin than on glass and PDMS surfaces. Cell detachment from the device occurred during long-term culture with the PDMS device but not with our gelatin device.

In conclusion, our gelatin device is suitable for cell culture operations and provides a good culture environment in terms of stiffness and permeability. In the future, we plan to apply this technology to vascular permeability testing and organ-on-a-chip or tumor-on-a-chip in combination with cancer cells.

## Figures and Tables

**Table 1:** Comparison of crosslinkers.

	Cost Effectiveness	Low Cytotoxicity	Ease of Observation
mTG	✓	✓	✓
Genipin	✓	-	-
GelMa	-	-	✓

## Data Availability

Not applicable.

## Supplementary Materials

The following supporting information can be downloaded at: https://www.mdpi.com/article/10.3390/mi14010107/s1, Figure S1: Illustration of the method used in this study for the determination of elastic modulus of a gelatin gel.; Figure S2: Optimization of microbial transglutaminase (mTG) concentration.; Figure S3: Inlet and outlet tube connections.; Figure S4: Measurement of the permeability of the gelatin gel via fluorescein or FITC-conjugated dextran diffusion.; Figure S5: Fibronectin fluorescence intensity distribution maps of Figure 5(Aii) Gelatin, (iv) PDMS, and (vi) APTES-GA-modified glass.; Figure S6: HUVECs co-cultured with NHDFs in PDMS or gelatin well devices form a capillary-like network.; Video S1: Vascular structure was constructed along the striped gelatin structure.
