# Supplementary material for: Fabrication of a Gelatin-Based Microdevice for Vascular Cell Culture
Source: Micromachines (Basel). 2022 Dec 30;14(1):107. doi: 10.3390/mi14010107 (PMC9860854; doi:10.3390/mi14010107)
Supplement: Supplementary file 1 [file micromachines-14-00107-s001.zip › Supplementary Materials1227.pdf]

Supplementary Materials

Fabrication of a gelatin-based microdevice for vascular cell culture

Satoko Sasaki[1], Tomoko Suzuki[1], Kyojiro Morikawa[2,3], Michiya Matsusaki[4], and Kae Sato[1]*

[1]Department of Chemical and Biological Sciences, Faculty of Science, Japan Women's University, 2-8-1 Mejirodai, Bunkyo, Tokyo 112-8681, Japan

[2]Institute of Nanoengineering and Microsystems, Department of Power Mechanical Engineering, National Tsing Hua University, No. 101, Section 2, Kuang-Fu Road, Hsinchu 300044, Taiwan, R.O.C.

[3]Collaborative Research Organization for Micro and Nano Multifunctional Devices, The University of Tokyo, 7-3-1 Hongo, Bunkyo, Tokyo 113-8656, Japan

[4]Division of Applied Chemistry, Graduate School of Engineering, Osaka University, 1-1 Yamadaoka, Suita, Osaka 565-0871, Japan

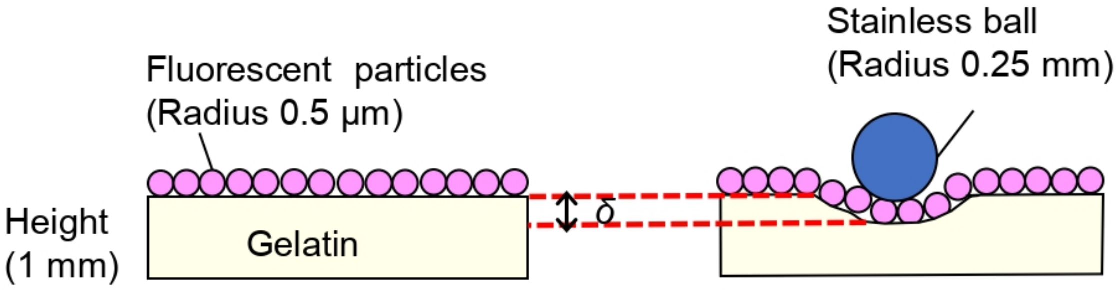

Figure S1. Illustration of the method used in this study for the determination of elastic modulus of a gelatin gel.

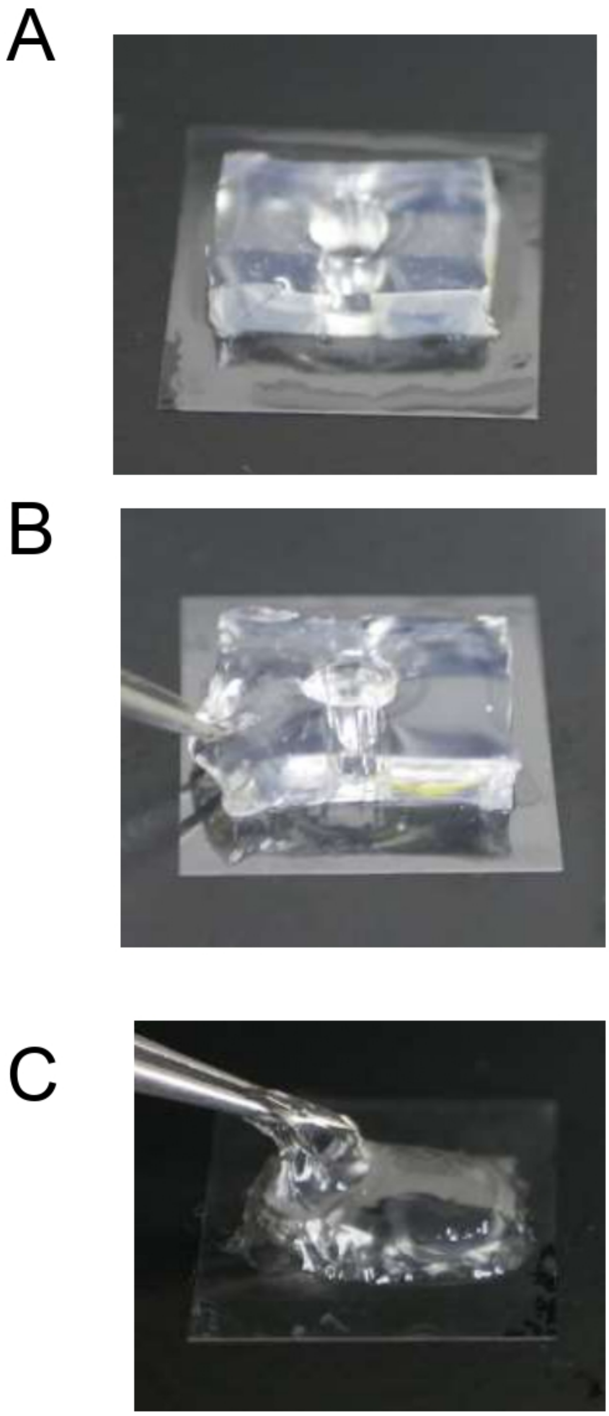

Figure S2. Optimization of microbial transglutaminase (mTG) concentration: photographs taken after 5 h crosslinking at 37°C (A) 1.0 U/mL, (B) 0.1 U/mL, and (C) 0.01 U/mL.

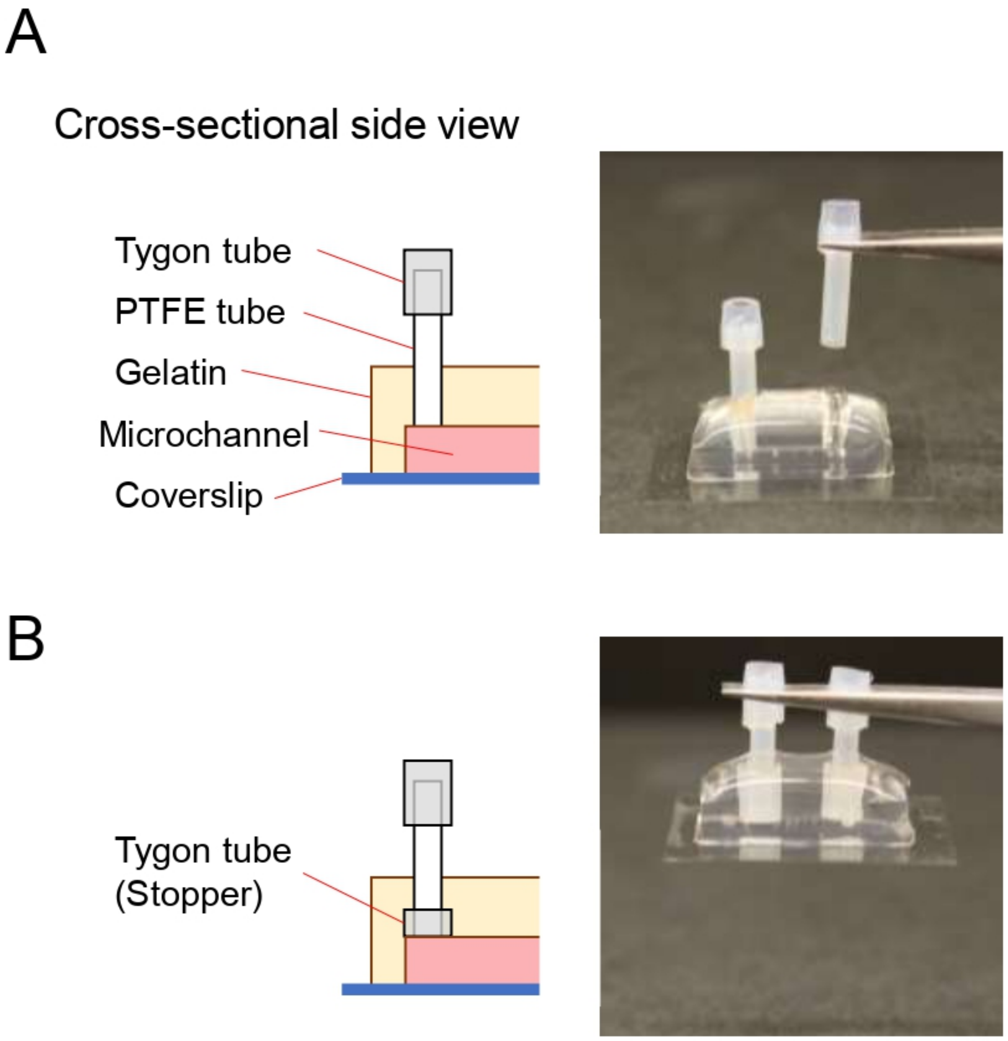

Figure S3. Inlet and outlet tube connections: (A) one end of the PTFE tube with the short Tygon tube; (B) Both ends of the PTFE tube are connected to the short Tygon tube. The Tygon tube embedded in gelatin gel plays a role in preventing the exit of the PTFE tube.

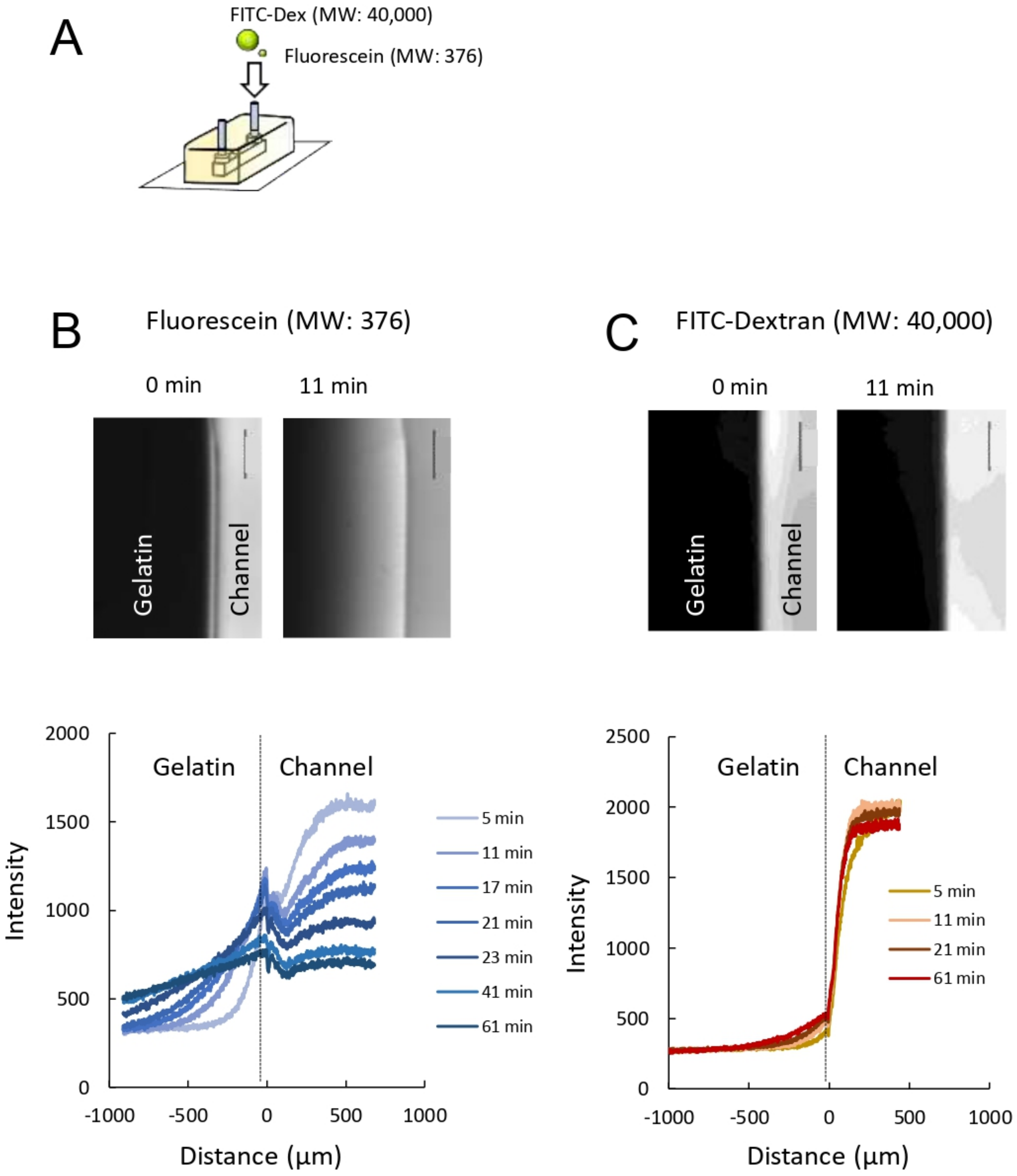

Figure S4. Measurement of the permeability of the gelatin gel via fluorescein or FITC-conjugated dextran diffusion: (A) Schematic diagram; fluorescence images of diffusion profile of (B) Fluorescein or (C) FITC-conjugated dextran (MW 40,000) across microchannels (scale bar: 500 µm) and line intensity profiles across the microchannel of gelatin microdevice at various time points.

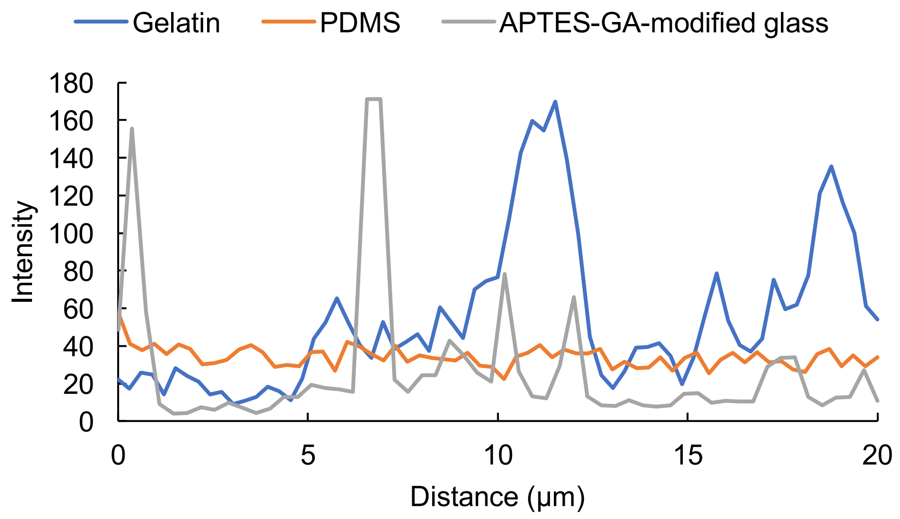

Figure S5. Fibronectin fluorescence intensity distribution maps of Figure 5A (ii) Gelatin, (iv) PDMS, and (vi) APTES-GA-modified glass.

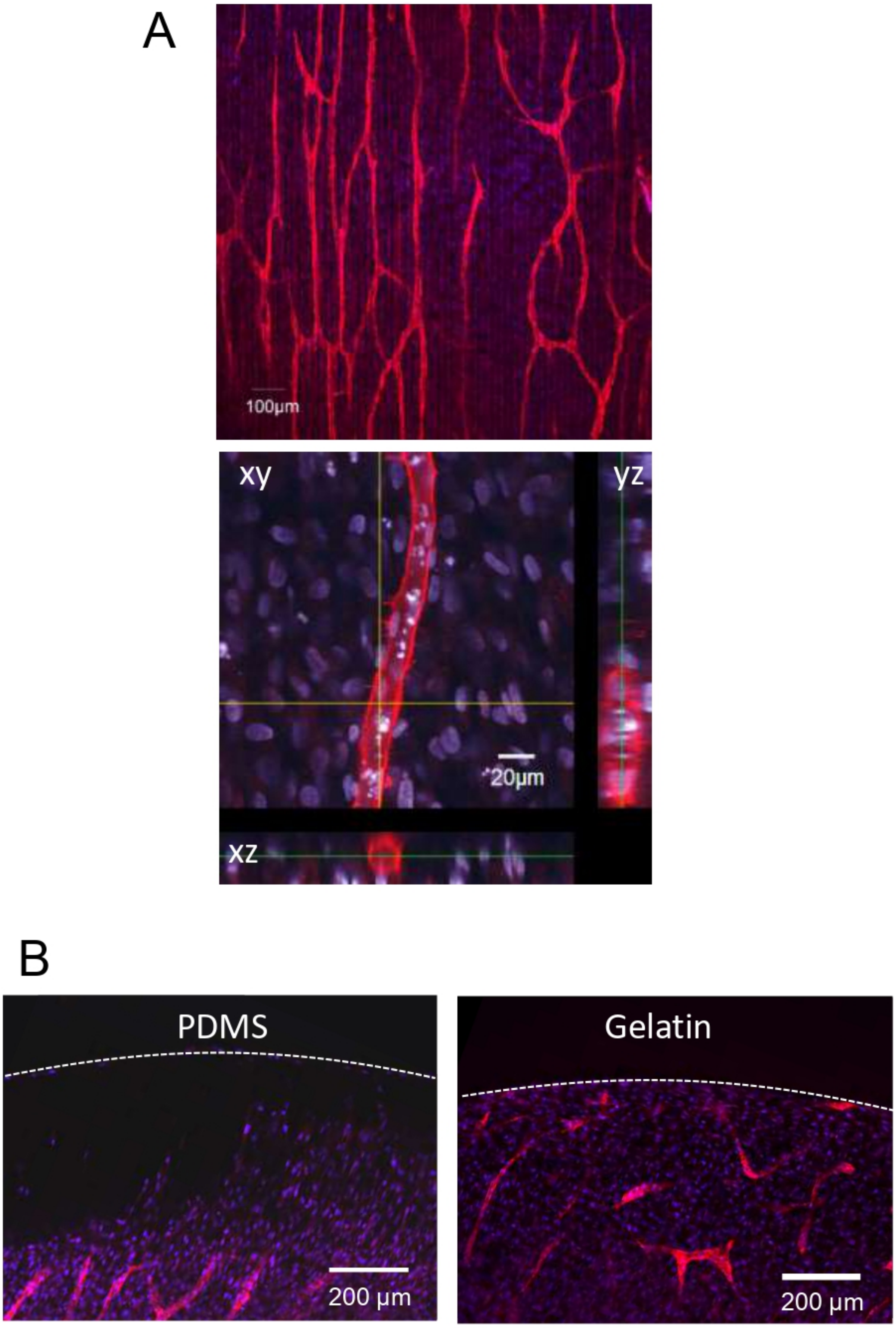

Figure S6. HUVECs co-cultured with NHDF in PDMS or gelatin well devices form a capillary-like network: (A) Confocal microscopy images of HUVEC capillary-like network (red) after 8 days of co-culture with NHDF (unlabeled) on stripe gelatin gel with a 6 μm height and 20 μm groove width; (B) Photograph of cells near the wall. The dotted line is the border between the PDMS or gelatin and the cell culture well. Cells were detached from the PDMS wall and gathered at the center of the well, but not from the gelatin well. Even after 8 days of incubation, the vascular network remains extensive throughout the gelatin well.
